# Indications of Selenium Protection against Cadmium and Lead Toxicity in Oilseed Rape (*Brassica napus* L.)

**DOI:** 10.3389/fpls.2016.01875

**Published:** 2016-12-15

**Authors:** Zhilin Wu, Xuebin Yin, Gary S. Bañuelos, Zhi-Qing Lin, Ying Liu, Miao Li, Linxi Yuan

**Affiliations:** ^1^Key Laboratory of Agri-Food Safety of Anhui Province, Scientific Observing and Experimental Station of Agricultural Environment of the Ministry of Agriculture – Laboratory of Quality and Safty Risk Assessment for Agricultural Products on Storage and Preservation of the Ministry of Agriculture, School of Plant Protection – School of Resources and Environment, Anhui Agricultural UniversityHefei, China; ^2^School of Earth and Space Sciences, University of Science and Technology of ChinaHefei, China; ^3^Jiangsu Bio-Engineering Research Centre of Selenium, Suzhou Institute for Advanced Study, University of Science and Technology of ChinaSuzhou, China; ^4^Institute of Advanced Technology, University of Science and Technology of ChinaHefei, China; ^5^San Joaquin Valley Agricultural Sciences Center, United States Department of Agriculture – Agricultural Research Service, ParlierCA, USA; ^6^Environmental Sciences Program and Department of Biological Sciences, Southern Illinois University Edwardsville, EdwardsvilleIL, USA; ^7^The Northwest of Anhui Province Station for Integrative Agriculture, Research Institute for New Rural Development, Anhui Agricultural UniversityHefei, China

**Keywords:** selenium, cadmium, lead, oxidative stress, oilseed rape (*Brassica napus* L.), antioxidant defense system

## Abstract

The present study investigated the beneficial role of selenium (Se) in protecting oilseed rape (*Brassica napus* L.) plants from cadmium (Cd^+2^) and lead (Pb^+2^) toxicity. Exogenous Se markedly reduced Cd and Pb concentration in both roots and shoots. Supplementation of the medium with Se (5, 10, and 15 mg kg^-1^) alleviated the negative effect of Cd and Pb on growth and led to a decrease in oxidative damages caused by Cd and Pb. Furthermore, Se-enhanced superoxide free radicals (${\text{O}}_{2}^{\bar{•}}$), hydrogen peroxide (H_2_O_2_), and lipid peroxidation, as indicated by malondialdehyde accumulation, but decreased superoxide dismutase and glutathione peroxidase activities. Meanwhile, the presence of Cd and Pb in the medium affected Se speciation in shoots. The results suggest that Se could alleviate Cd and Pb toxicity by preventing oxidative stress in oilseed rape plant.

## Introduction

Protection against the accumulation and toxic effects of heavy metals on humans, animals, and plants are topics of current interest due to the increasing emission of pollutants resulting from industries and anthropogenic activities (Hall, 2002; Filek et al., 2008, 2010; Cartes et al., 2010; Feng et al., 2013). Cadmium (Cd) and lead (Pb) are harmful heavy metals pollutants in soils and water, and pose health risk for humans through the food chain due to their plant-availability in soils (He et al., 2004; Belkhadi et al., 2010; Filek et al., 2010; Gallego et al., 2012; Lin et al., 2012; Saidi et al., 2014). The deleterious effects of Cd and Pb to plants and other organisms have previously been investigated (Belzile et al., 2006; Tang et al., 2015). Plant species and varieties show large differences in heavy metals tolerance, from metal high-sensitivity to metal hyperaccumulation and hypertolerance phenotypes in plants (Metwally et al., 2003; Popova et al., 2009; Belkhadi et al., 2010; Saidi et al., 2014). To avoid phytotoxicity of heavy metals, plants adopt different defense strategies including the production of metallothioneins (MTs) and phytochelatins (PCs) (Lin et al., 2012; Saidi et al., 2014; Qing et al., 2015). One of the protective mechanisms employed is the induction of the antioxidant defense system, which involves the sequential and simultaneous action of a number of enzymes such as peroxidase (POD), catalase (CAT), superoxide dismutase (SOD), ascorbate peroxidase (APX), and non-enzymatic scavengers such as glutathione (GSH) and ascorbate (AsA). The latter are responsible for scavenging excessively accumulated reactive oxygen species (ROS) in plants under stress conditions (Lin et al., 2012; Saidi et al., 2014; Qing et al., 2015). Cadmium and Pb could cause oxidative damage to plants, directly or indirectly through the generation of ROS (Lin et al., 2012; Khan et al., 2015). Others have found that Cd and Pb cause toxicity, which affects plant growth, development, and metabolisms, as well as other physiological processes, including inhibition of seed germination, alteration of enzymatic function, impaired photosynthesis, membrane damage (Schützendübel and Polle, 2002; Shekhawat et al., 2010; Feng et al., 2013; Irfan et al., 2014). Therefore, it is of vital importance to develop reliable approaches such as plant breeding and chemical regulators to prevent heavy metals accumulation in plants. Among them, the application of chemical regulators to alleviate heavy metal toxicity and reduce plant heavy metal uptake in farmland contaminated with heavy metals, might be a practical and cost-effective strategy for sustainable utilization of natural resources and production of safe, high-quality agro-products. (Lin et al., 2012; Noriega et al., 2012; Cao et al., 2013; Ali et al., 2015).

Selenium (Se) is an essential trace element for humans and animals, and some findings suggest that Se may be a beneficial element, which plays a novel role in plant biology for innovative crop production (Trumble et al., 1998; Rayman, 2000; Terry et al., 2000; Ellis and Salt, 2003; Hartikainen, 2005; Pilon-Smits et al., 2009; Zhu et al., 2009; Bañuelos et al., 2011; Hatfield et al., 2014; Wu et al., 2015). Selenium at low concentrations exerts positive effects for plants such as promoting growth, increasing antioxidative capacity, improving yield and quality, and delaying ripening and senescence (Hartikainen et al., 2000; Xue et al., 2001; Turakainen et al., 2004; Broadley et al., 2010; Pukacka et al., 2011; Pezzarossa et al., 2012). Applying Se fertilizer as base fertilizer or foliar spray has been used to increase the Se content in the edible portion of crops and to simultaneously counteract the detrimental effects of diverse environmental stresses, such as heavy metals, water, drought, salt, high temperature, UV radiation, senescence, pathogens, and insect pests (Hartikainen et al., 2000; Pennanen et al., 2002; Hanson et al., 2003; Djanaguiraman et al., 2005, 2010; Germ et al., 2005; Broadley et al., 2010; Yao et al., 2010; Zembala et al., 2010; Hasanuzzaman and Fujita, 2011; Hasanuzzaman et al., 2011; Pukacka et al., 2011; Kumar et al., 2012; Pezzarossa et al., 2012; Feng et al., 2013; Ardebili et al., 2014; Ahmad et al., 2016; Kaur et al., 2016; Wu et al., 2016).

Recently, the beneficial role of Se in alleviation of heavy metal- induced oxidative stress has been well established (Filek et al., 2008, 2010; Han et al., 2015). The vital role of Se in antioxidant protection from heavy metal stress has been observed in different biological systems (Yathavakilla and Caruso, 2007; Kumar et al., 2012; Lin et al., 2012; Malik et al., 2012) including some crop plants (Lin et al., 2012; Mroczek-Zdyrska and Wojcik, 2012; Mozafariyan et al., 2014). Others have recognized that Se can detoxify many toxic heavy metals such as Cd, Pb, chromium (Cr), and arsenic (As) in plants (Shanker et al., 1996a,b; Srivastava et al., 1998; Feng et al., 2013; Saidi et al., 2014; Qing et al., 2015). Though many studies have demonstrated that external Se may act as a chemical regulator to reduce heavy metal-induced oxidative stress, a better understanding of the mode of action of Se in prevention of heavy metal accumulation in plants should be achieved. Moreover, the possible mechanisms of the Se-enhanced tolerance and/or resistance of plants to Cd and Pb stresses remain unclarified. The present study was undertaken to investigate the potential role of external Se in modulating Cd/Pb-induced oxidative stress and Cd/Pb uptake/translocation in oilseed rape plant. We aim to provide a basis for developing strategies to reduce risks associated with Cd and Pb toxicity and maintaining sustainable crop production.

## Materials and Methods

### Plant Material, Soil Collection, and Experimental Designs

Potting compost was used in the experiment and brought from Huai’an Hongyang Agricultural Technology Co. The soil’s main properties were as follows: N+P_2_O_5_+K_2_O ≥ 2%, contents of organic matter ≥40%. Eight kilogram “HONGYANG” potting compost soil was weighed and loaded into a plastic pot (10 L, 20 cm height). The pot culture experiments were conducted in a greenhouse at the Suzhou Campus, the University of Science and Technology of China, Suzhou, China, under natural light conditions. Oilseed rape seeds (*Brassica napus* L. cv. Wanyou 18) were germinated and the seedlings were grown in the soil. After 6 weeks of growth, morphologically similar and healthy seedlings were selected and transplanted into the pots. The experiment had a completely randomized design with three replicates. Each replicate consisted of five plants per pot, and only two plants from each pot were selected for final harvest. The seedlings were allowed to grow for 15 days in the pots for acclimatization. After 15 days of growth, treatment were applied as follows: (a) Se as sodium selenite alone (0, 1, 5, 10, 15, 20 mg kg^-1^), (b) Cd as cadmium chloride alone (1 or 5 mg kg^-1^), and combined with Se as sodium selenite (0, 1, 5, 10, 15, 20 mg kg^-1^), respectively, (c) Pb as lead acetate alone (300 or 500 mg kg^-1^), and combined with Se as sodium selenite (0, 1, 5, 10, 15, 20 mg kg^-1^), respectively. The Se concentrations were selected based upon a previous study (Saidi et al., 2014), while Pb and Cd concentrations were selected on the basis of national soil environmental quality standard (GB15618-1995) and the current levels of soil metal pollution in China. Plant samples were collected 40, 60, 80, and 100 days after treatment at seedling stage, immediately immersed in liquid nitrogen and stored frozen at -80°C for further analyses or directly used for various biochemical assays.

### Determination of Se, Pb, and Cd Concentrations in Oilseed Rape Plants

The sample preparation and the measurement of total Se were performed according to the methods of Lin et al. (2012) and Saidi et al. (2014). After plant samples were thoroughly washed with water, roots were soaked in 20 mM EDTA for 15 min to remove adsorbed metals on the root surfaces and rinsed with distilled water (Barton and Abadia, 2006; Su et al., 2014), plant samples were oven-dried at 50°C for 48 h and ground to pass through a 0.2 mm sieve. 2.0 g samples were weighed into a 50 mL conical flask and 10 mL of concentrated HNO_3_ and HClO_4_ (4:1, v/v) were added to each flask. After a series of temperature programming and acid digestion at 100°C, HNO_3_ was expelled completely and the volume of solution was approximately 2 mL. Then 5 mL HCl (12 M) was added to reduce Se (VI) to Se (IV) for 3–4 h and the digestion solution was brought up to 25 mL for Se analysis. The total Se concentration was determined by Hydride Generation-Fluorescence Spectrometry (HG-AFS 9230; Beijing Titan Instrument Co., China).

Concentrations of Pb and Cd were measured by the method of Determination of Lead in Foods (China GB 5009.12-2010 National Food Safety Standard) and Determination of Cadmium in Foods (China GB 5009.15-2003 National Food Safety Standard) with some modifications. 1.0 g samples were digested in fired porcelain crucibles and dry ashed in a muﬄe furnace 6–8 h at 500°C, and then cooled. The ash content was dissolved in HNO_3_ (0.5 M) and the digestion solution was diluted to 25 mL prior to Pb and Cd analysis. Concentrations of Cd and Pb in the roots and shoot samples were analyzed by atomic absorption spectroscopy (PerkinElmer AA-700, Shanghai PerkinElmer Inc., China) with graphite furnace.

### Assay for Antioxidative Enzyme Activities

To determine enzyme activity after different treatments, fresh whole shoots of oilseed rape were homogenized in 8 mL 50 mM PBS (pH 7.8) using a prechilled mortar and pestle, and subsequently centrifuged at 10,000 × *g* for 15 min at 4°C. The supernatant was used for the assays of antioxidative enzyme activity. SOD activity was determined as described by Lin et al. (2012) and Saidi et al. (2014). Glutathione peroxidase (GPx) activity was measured using modification of the procedure by Saidi et al. (2014) and Ali et al. (2015).

### Determination of H_2_O_2_ and ${\text{O}}_{2}^{\bar{•}}$ and Lipid Peroxidation

One gram fresh shoots of oilseed rape plants was ground together with phosphate buffer (pH 7.0) and a small quantity of quartz sand in liquid nitrogen. The homogenate was centrifuged at 10000 × *g* for 15 min and the supernatant was used for determination of hydrogen peroxide (H_2_O_2_) and superoxide anion (${\text{O}}_{2}^{\bar{•}}$). Hydrogen peroxide production rate was determined with the method by Lin et al. (2012) and Saidi et al. (2014). Superoxide anion production rate was determined with the method by Lin et al. (2012) and Qing et al. (2015). The level of lipid peroxidation in plant tissues was determined as 2-thiobarbituric acid (TBA) reactive metabolites, mainly malondialdehyde (MDA) with the method by Lin et al. (2012) and Qing et al. (2015).

### Chemical Speciation Analysis of Se in Shoots of Oilseed Rape Plant

Analysis of Se speciation was performed according to the method by Bañuelos et al. (2011) and Yuan et al. (2013). The whole shoots were extracted with 100 mM Tris-HCl buffer (pH 7.5) in an ultrasonic tank for 10 min and then enzyme Protease XIV was then added to shake for 24 h at 37°C. After extraction, the mixture was centrifuged at 10000 × *g* for 30 min at 4°C. The supernatant was collected and filtered through a 0.22 mm filter prior to Se speciation analysis. A Hamilton PRP X-100 anion exchange column (4.1 mm × 250 mm × 10 μm) was used to separate different Se compounds. The mobile phase was 40 mM NH_4_H_2_PO_4_ (pH 6.0) with a flow rate of 1 mL min^-1^. The eluent from the column was mixed with concentrated HCl and then passed through the UV unit. 1.2% NaBH_4_ in 0.1 mol L^-1^ NaOH was added after the UV unit. Argon was used as carrier gas to transfer H_2_ from the gas liquid separator through the dryer into the AFS detector. The dryer gas was nitrogen with a flow rate of 3 L min^-1^. Then, Se species in shoots of oilseed rape plant were detected by LC-UV-HG-AFS system (Beijing Jitian instruments Co., Ltd, China).

### Statistical Analysis

The experiment was replicated three times under the same conditions. All statistical analyses were performed using the SPSS software version 13.0 (SPSS Inc., Chicago, IL, USA). Significant differences between treatment effects were determined by one way ANOVA followed by Duncan’s multiple range test for multiple comparisons with statistical significance of *p* ≤ 0.05.

## Results

### Effects of Exogenous Se on Pb and Cd Accumulation in Oilseed Rape Seedlings

**FIGURE 1:**
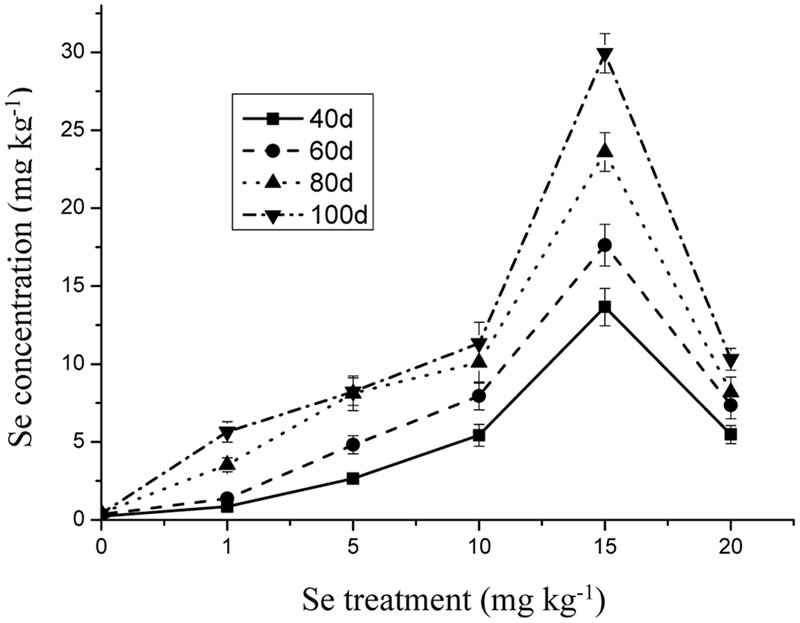
**Concentration of Se in shoots of oilseed rape treated with soil Se in different periods.** Average concentrations (Dry biomass weight, DBW) provided with error bars representing the standard error. (*p* ≤ 0.05).

**FIGURE 2:**
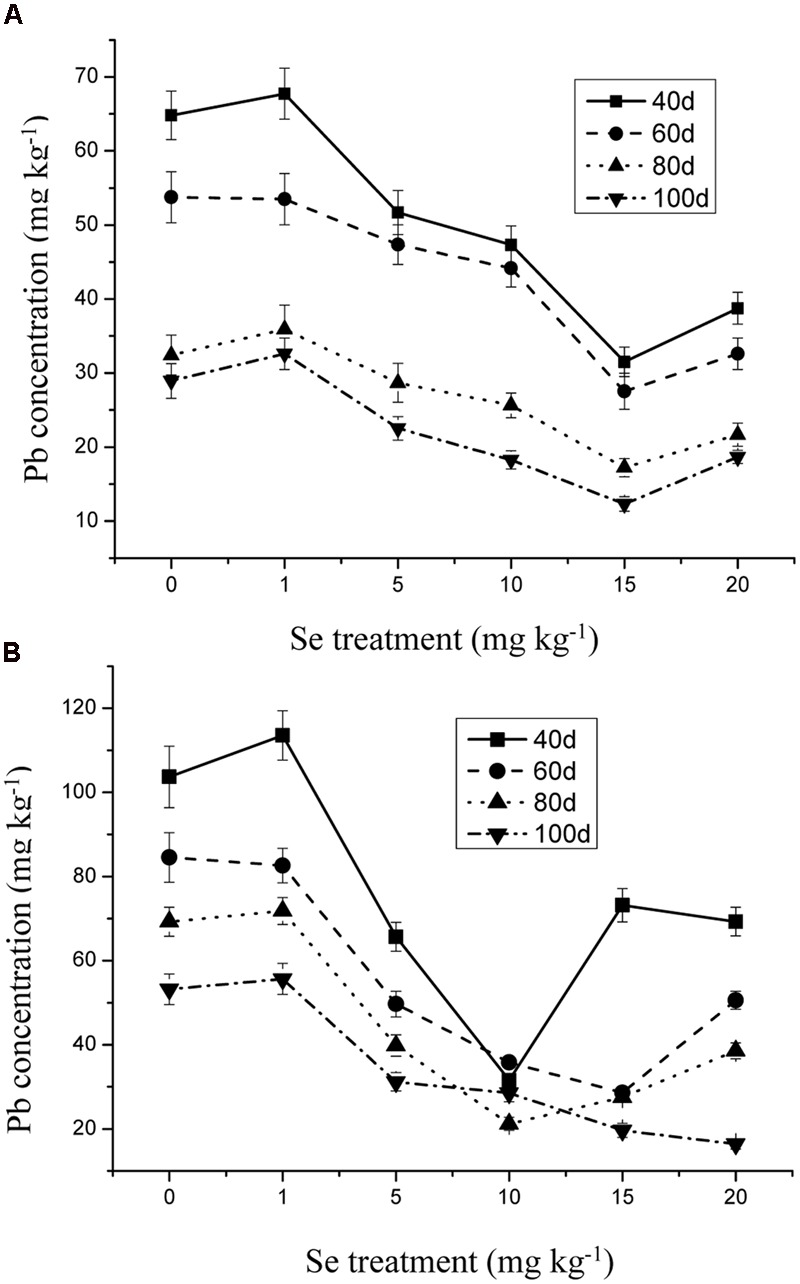
**(A)** Concentration of Pb in shoots of oilseed rape treated with soil Se and 300 mg kg^-1^ Pb in different periods. **(B)** Concentration of Pb in shoots of oilseed rape treated with soil Se and 500 mg kg^-1^ Pb in different periods. Average concentrations (Dry biomass weight, DBW) provided with error bars representing the standard error. (*p* ≤ 0.05).

**FIGURE 3:**
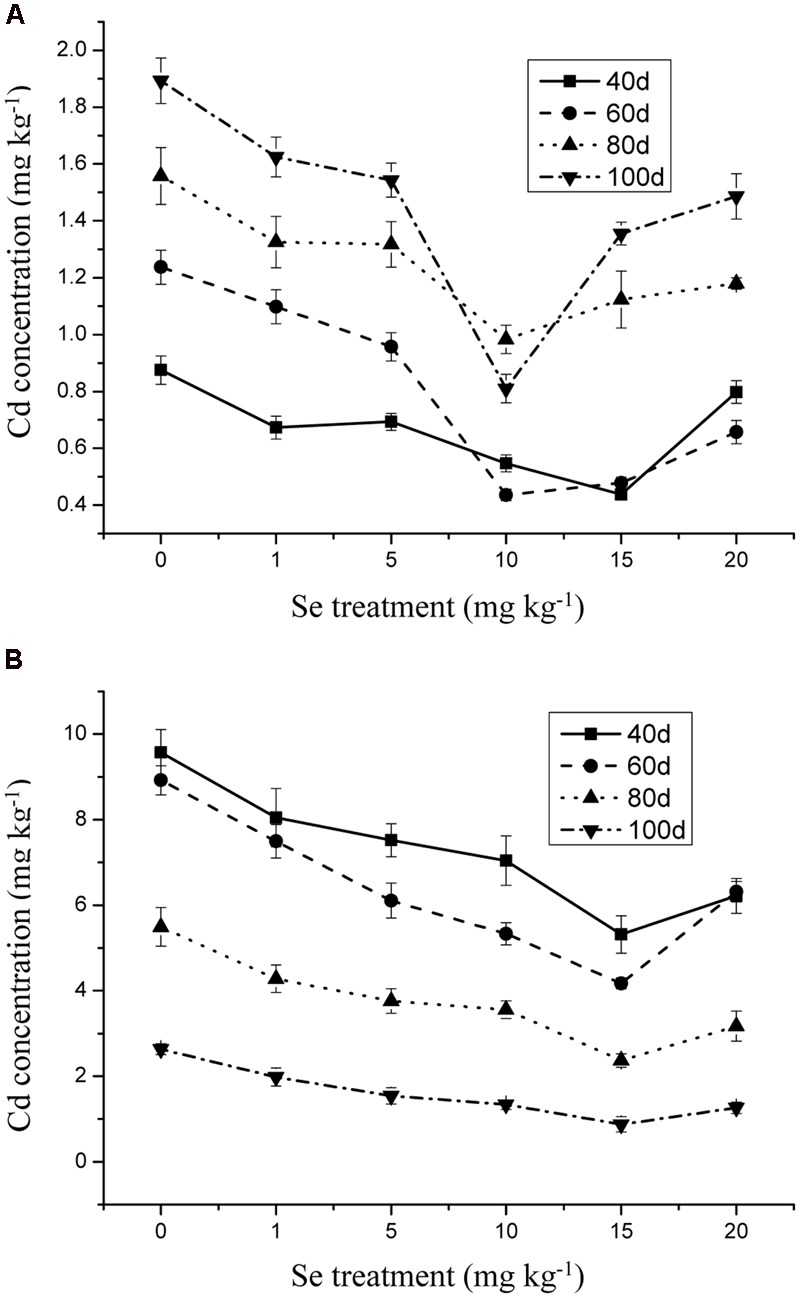
**(A)** Concentration of Cd in shoots of oilseed rape treated with soil Se and 1 mg kg^-1^ Cd in different periods. **(B)** Concentration of Cd in shoots of oilseed rape treated with soil Se and 5 mg kg^-1^ Cd in different periods. Average concentrations (Dry biomass weight, DBW) provided with error bars representing the standard error. (*p* ≤ 0.05).

The Se concentration in the shoots increased significantly with increasing Se level in the soil from 0 to 15 mg kg^-1^ in the soil (**Figure 1**) (*p* ≤ 0.05). However, the Se concentration in the plant tissue was reduced to 10.31 mg kg^-1^ when Se addition was from 15 to 20 mg kg^-1^. In addition, Se at 10 mg kg^-1^ significantly decreased the Cd and Pb concentrations in the oilseed rape (**Figures 2** and **3**) (*p* ≤ 0.05).

**FIGURE 4:**
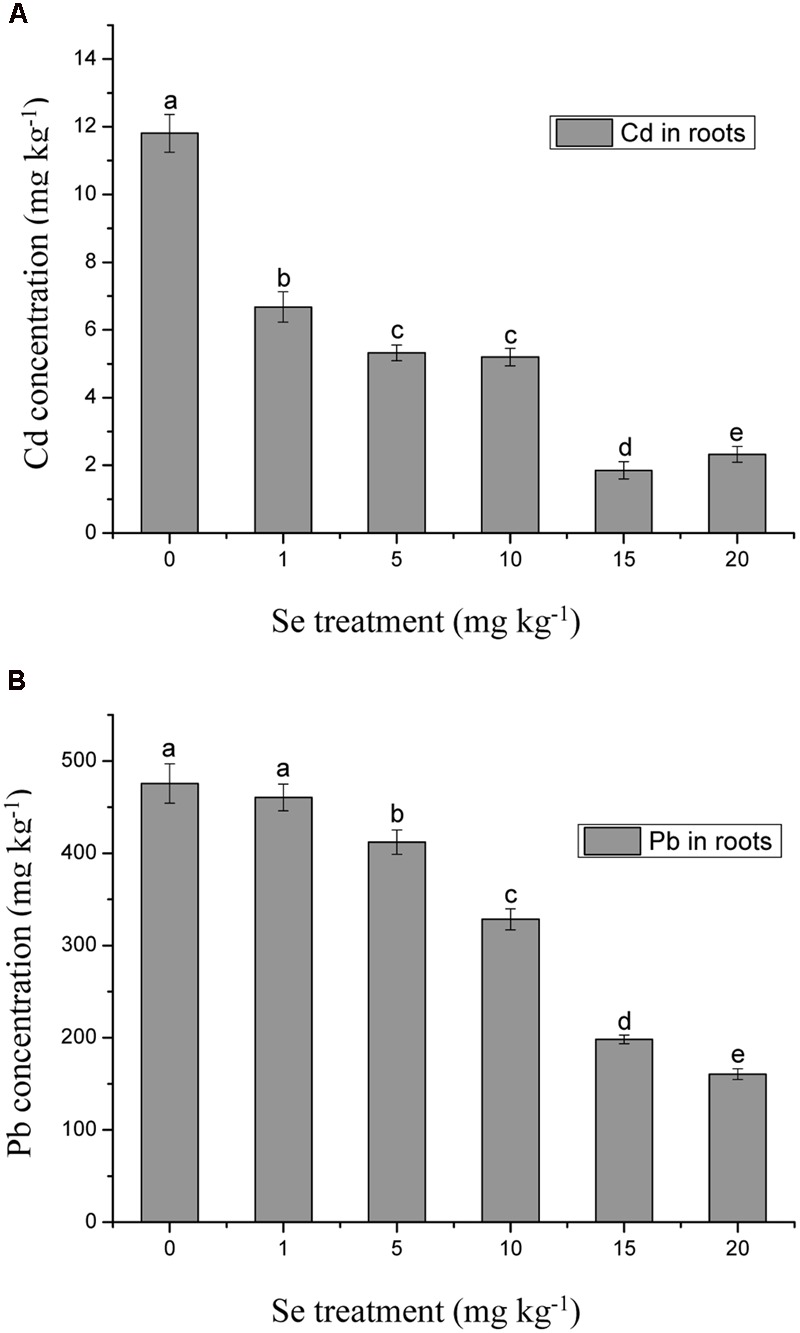
**(A)** Concentration of Cd in roots of oilseed rape treated with soil Se and 5 mg kg^-1^ Cd after a planting time of 60 days. **(B)** Concentration of Pb in roots of oilseed rape treated with soil Se and 500 mg kg^-1^ Pb after a planting time of 60 days. Average concentrations (Dry biomass weight, DBW) provided with error bars representing the standard error. Different letters mean significance of difference between the treatments (*p* ≤ 0.05).

The concentrations of Cd and Pb in the roots significantly decreased with increasing Se treatment level, suggesting that Se inhibited Cd and Pb accumulation in roots and consequently further reduced heavy metal toxic effects in shoots (**Figure 4**). Compared with the control, the Se treatment of 15 mg kg^-1^ reduced the root Cd concentration by 84.3% (**Figure 4A**), while the root Pb concentration was reduced by 66.3% at the Se treatment of 20 mg kg^-1^ (**Figure 4B**) (*p* ≤ 0.05). The reduction of Cd and Pb concentrations in roots was much greater than in the shoots (*p* ≤ 0.05).

### Effects of Se on Antioxidant Enzymes Activities

**FIGURE 5:**
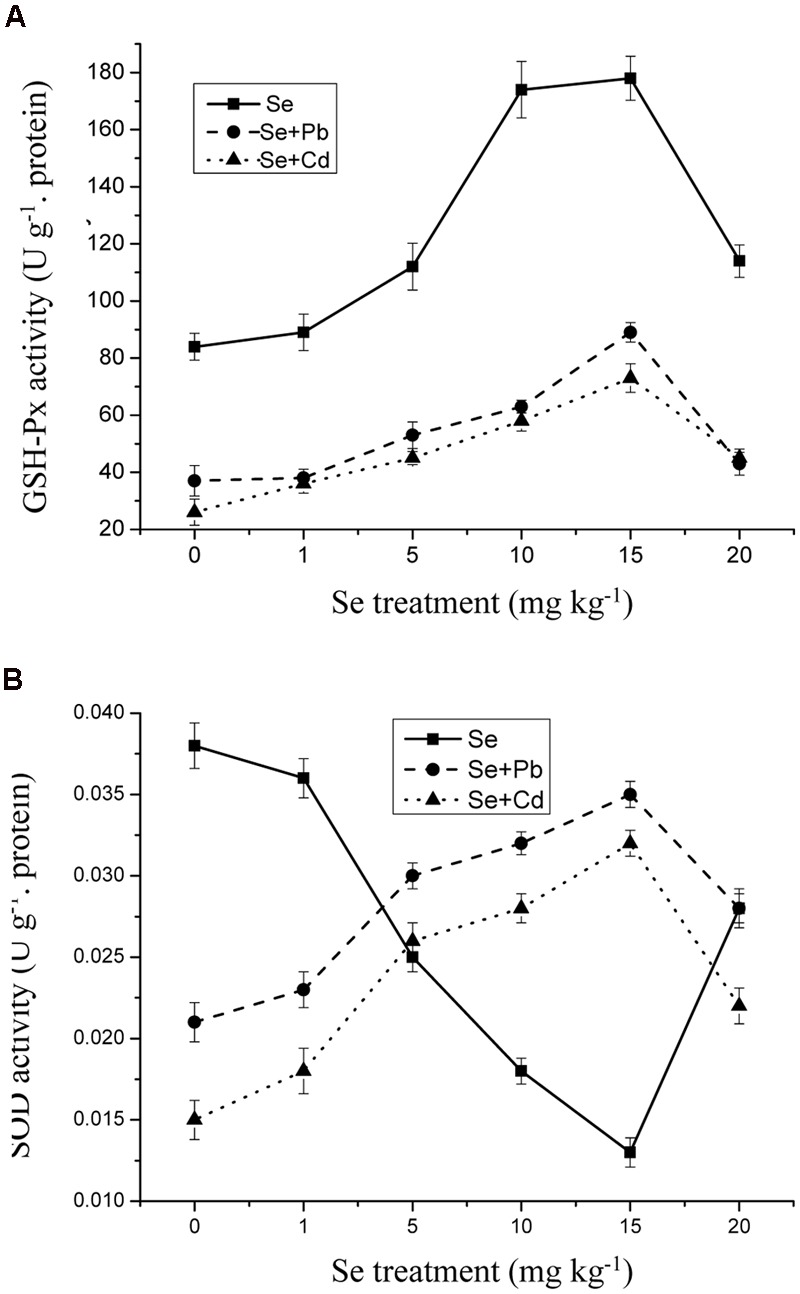
**(A)** The activity of glutathione peroxidase (GSH-Px) in shoots of oilseed rape treated with soil Se, Se+5Cd, or Se+500Pb after a planting time of 60 days. **(B)** The activity of superoxide dismutase (SOD) in shoots of oilseed rape treated with soil Se, Se+5Cd, or Se+500Pb after a planting time of 60 days. Average values (Fresh biomass weight, FBW) provided with error bars representing the standard error. (*p* ≤ 0.05).

Selenium supplementation resulted in a significant decrease in antioxidative enzymes activities in the shoots upon Cd and Pb exposure. Moreover, SOD and GPx activities in the shoots were strongly affected by the Se treatment of 15 mg kg^-1^. However, a significant decrease in GPx and SOD activities in the shoots was observed at 20 mg kg^-1^ Se (**Figures 5A,B**) (*p* ≤ 0.05).

### Effects of Se on Malondialdehyde, H_2_O_2_, and ${\text{O}}_{2}^{\bar{•}}$

**FIGURE 6:**
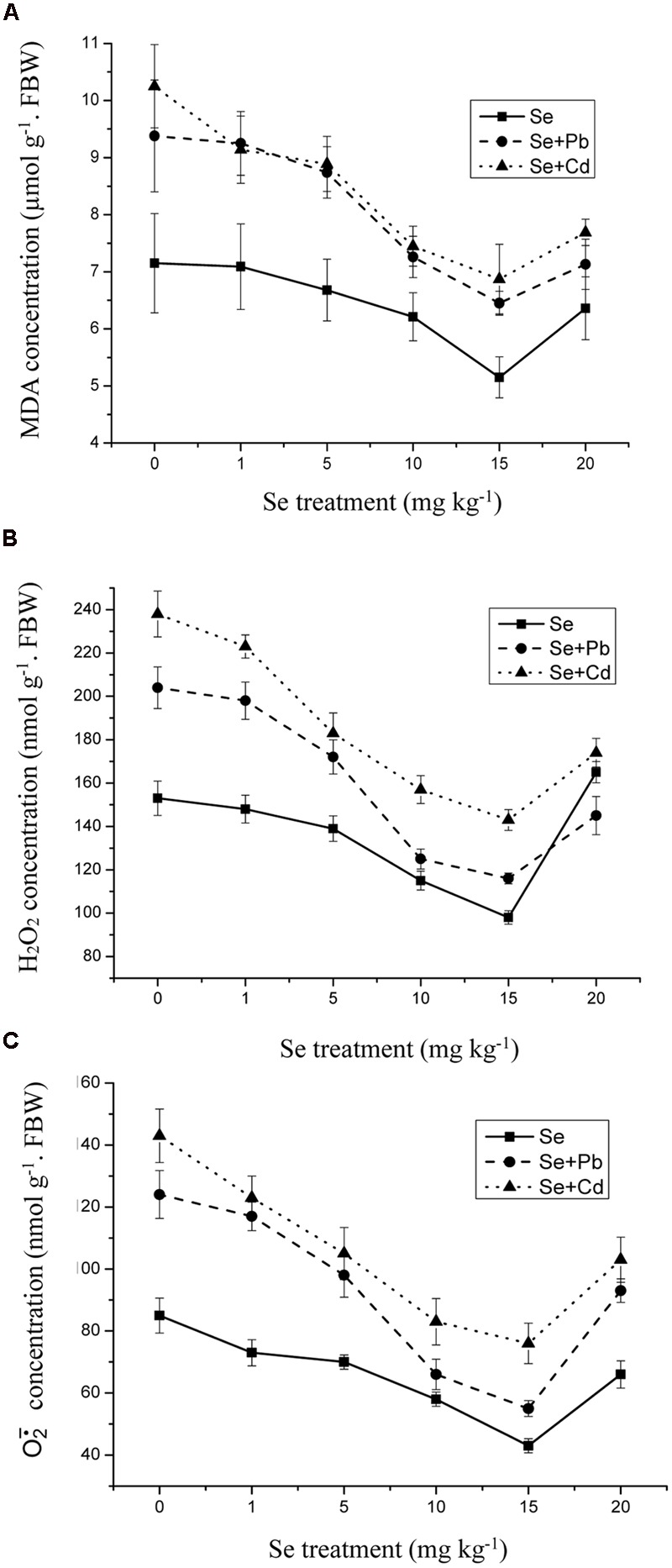
**(A)** The concentration of malondialdehyde (MDA) in shoots of oilseed rape treated with soil Se, Se+5Cd, or Se+500Pb after a planting time of 60 days. **(B)** The concentration of hydrogen peroxide (H_2_O_2_) in shoots of oilseed rape treated with soil Se, Se+5Cd, or Se+500Pb after a planting time of 60 days. **(C)** The productive rate of superoxide anion (${\text{O}}_{2}^{\bar{•}}$) in shoots of oilseed rape treated with soil Se, Se+5Cd, or Se+500Pb after a planting time of 60 days. Average values (Fresh biomass weight, FBW) provided with error bars representing the standard error. (*p* ≤ 0.05).

An accumulation of lipid peroxides is indicative of enhanced production of ROS. The level of MDA, one of the major TBA reactive metabolites, increased by 31% in the 500 mg kg^-1^ Pb-treated shoots and by 43% in the 5 mg kg^-1^ Cd-treated shoots compared to control (**Figure 6A**) (*p* ≤ 0.05). Furthermore, the Se treatment of 1 mg kg^-1^ did not significantly influence the MDA level, while 15 mg kg^-1^ Se decreased the lipid peroxidation by 33% compared to control. In addition, the Se treatment had a significant effect against lipid peroxidation in the shoots, especially in the Cd treated group. The same tendency also appeared on the change of H_2_O_2_ levels and ${\text{O}}_{2}^{\bar{•}}$ activities. The addition of Se at 10–15 mg kg^-1^ lessened the generation of H_2_O_2_ levels and ${\text{O}}_{2}^{\bar{•}}$ activities induced by Pb and Cd. (**Figures 6B,C**).

### Speciation of Se in Plant Tissues

The extraction efficiency of total Se was between 8 and 29% in the oilseed rape shoots (**Table 1**). SeCys2 and SeMeCys were not found in shoots of oilseed rape plant under current detection conditions. SeMet was the major form of Se detected and accounted for 8–27% (*p* ≤ 0.05). Moreover, approximately 5% Se (IV) was detected in the Se+Pb treatments.

**Table 1:** Selenium (Se) speciation in shoots of oilseed rape treated with soil Se, Se+Cd, or Se+Pb after a planting time of 60 days (Dry biomass weight, DBW).

Treatments (mg kg^-1^)	Total Se (mg kg^-1^)	SeMet (mg kg^-1^)	Se^4+^ (mg kg^-1^)	SeMet as percentage of total Se	Se^4+^ as percentage of total Se
15Se	29.95 ± 0.12a	2.584 ± 0.05a	0	8.63%	0
15Se+300Pb	28.63 ± 0.18a	5.473 ± 0.04b	2.783 ± 0.06	19.12%	9.72%
15Se+500Pb	25.92 ± 0.14a	4.298 ± 0.08b	1.225 ± 0.03	16.60%	4.72%
15Se+1Cd	26.58 ± 0.25a	6.841 ± 0.05b	0	25.74%	0
15Se+5Cd	19.89 ± 0.15b	5.447 ± 0.06b	0	27.36%	0

*All data show the means*+*SE of three replicates. Different letters mean significance of difference between the treatments (*p* ≤ 0.05).*

## Discussion

Heavy metal contamination is a major concern worldwide, hence strategies for protecting human and environmental health from heavy metals contaminants are of high priority (Gallego et al., 2012; Lin et al., 2012; Qing et al., 2015). The breeding of plant cultivars and the application of chemical regulators in order to reduce heavy metal uptake and accumulation may alleviate heavy metals toxicity in plants and improve crop quality by increasing the concentrations of beneficial trace elements (Grant et al., 2008; Feng et al., 2013; Ali et al., 2015; Kaur et al., 2016). The beneficial role of Se in alleviation of heavy metal-induced oxidative stress is well established, and the alleviating effects of Se on heavy metal toxicity to plants by decreasing uptake of Hg, As, Cd, Cr, and Pb have been reported (Feng et al., 2013; Saidi et al., 2014). Moreover, in the presence of phytotoxic levels of heavy metals, proper supplementation with Se may alleviate growth inhibition (Filek et al., 2008, 2010; Feng et al., 2013). The present study demonstrates the beneficial role of Se in reducing Cd and Pb uptake and toxicity by preventing oxidative stress in oilseed rape plants (**Figures 2**–**4**).

Selenium, an essential element for animals and humans, has also been found to be beneficial to plants (Ellis and Salt, 2003; Wu et al., 2015). However, Se often exerts a dual effect on plant growth. At low doses, it can stimulate the growth of plants and counteract many types of environmental stresses, including heavy metals, whereas at high levels, it can be toxic and cause oxidative damage to plants and other biota (Lin et al., 2012; Feng et al., 2013; Saidi et al., 2014; Qing et al., 2015). Although most higher plants do not require Se, the supplementation of Se fertilizers with sodium selenate or sodium selenite can positively affect the whole food chain from soil to plants, animals, and humans (Hartikainen, 2005; Lin et al., 2012; Feng et al., 2013; Saidi et al., 2014; Qing et al., 2015). Selenium is primarily taken up from the soil by plants as selenate (SeO_4_^2-^) or selenite (SeO_3_^2-^), which is metabolized through the sulfate assimilation pathway and incorporated into multiple organic Se compounds (Hartikainen, 2005; Lin et al., 2012; Feng et al., 2013; Saidi et al., 2014; Qing et al., 2015). He et al. (2004) found that the addition of selenite to lettuce (*Lactuca sativa* L.) plants subjected to Pb and Cd, significantly decreased the accumulation of these heavy metals and at the same time enhanced the uptake of some beneficial elements including Se. In this study, the Se concentrations of 15 mg kg^-1^ was the selective dosage for protecting oilseed rape from Cd and Pb toxicity. Moreover, Cd and Pb concentrations in the roots showed lower accumulation than that in the shoots, which may indicate that Se exerted antagonistic effects on Cd- and Pb-induced stresses in the roots (**Figures 2**–**4**). In addition, plants have a range of potential mechanisms that may be involved in the detoxification of heavy metals. The mechanisms appear to be involved primarily in avoiding the buildup of toxic concentrations at sensitive sites within roots, thus, preventing plants from damaging effects (Lin et al., 2012; Feng et al., 2013; Saidi et al., 2014; Qing et al., 2015). The mechanisms induced by Se might be related to the inhibition of uptake and translocation of heavy metals from the roots to shoots and/or the speciation transformation of Cd and Pb to non-toxic species (Lin et al., 2012; Feng et al., 2013; Saidi et al., 2014; Qing et al., 2015). For example, Shanker et al. (1996c) suggested that Se possessed a strong ability to combine with heavy metals to form non-toxic Se-metal complexes in the soil-root environment. The current results provide strong evidence that Se can effectively alleviate Cd/Pb-induced growth inhibition, suggesting that, again, low levels of Se can exert beneficial effects on plants.

Oxidative stress is a key part of abiotic and biotic stresses (Feng et al., 2013; Saidi et al., 2014). This mechanism is caused by a serious cell damage by production of ROS, including ${\text{O}}_{2}^{\bar{•}}$ and H_2_O_2_, and antioxidative enzymes, which leads to dramatic physiological disorders. Cd/Pb-induced oxidative stress, reflected by increased MDA content, could be attributed to the increase of lipid peroxidation in plant cells (Lin et al., 2012; Saidi et al., 2014; Qing et al., 2015). The decreases in ${\text{O}}_{2}^{\bar{•}}$ and H_2_O_2_ with addition of Se, suggest a disturbance of the ROS reaction chain, which diminishes the damage to the lipids of the plant cell membranes (Lin et al., 2012; Saidi et al., 2014; Qing et al., 2015). In this study, selenium supplementation has been shown to reduce Cd/Pb-induced oxidative stress as reflected by reduced ${\text{O}}_{2}^{\bar{•}}$, H_2_O_2_, and MDA (**Figure 6**). Therefore, our results suggest that the amelioration effects of Se on heavy metal stress are partly due to the improvement of scavenging capability of ROS, decrease in lipid peroxidation, and change in the membrane physicochemical characteristics, which increase plant cell integrity.

To scavenge ROS in cells and to avoid oxidative damage when exposed to abiotic or biotic stress, plants develop enzymatic, and non-enzymatic systems (Lin et al., 2012; Feng et al., 2013; Saidi et al., 2014; Qing et al., 2015). The present study has provided strong evidence of the protective role of Se against oxidative stress resulting from Cd/Pb stress (**Figures 5** and **6**). The protective role appears to be responsible for beneficial effects of Se in terms of activation of shoot SOD and GPx during the entire treatment. However, supplementation of the medium with Se decreased Cd/Pb-induced high shoot SOD and GPx activity (**Figure 5**). The response of the SOD enzyme is closely related with Se, which was complicated when plants are stressed under various adverse environmental conditions in the presence of Se. At low stress levels, the antioxidative capacity in a plant should be sufficient to support the normal growth of the plant, while under a severe stress from exposure to heavy metals, high levels of ROS will be produced, and SOD activity is required. The enhancement in SOD activity might suggest the excess production of ${\text{O}}_{2}^{\bar{•}}$ due to the toxicity of Se. The ROS production and quenching can thus be well controlled by antioxidants, resulting in the spontaneous reduction of ${\text{O}}_{2}^{\bar{•}}$ and a reduced requirement for SOD (Shekhawat et al., 2010; Lin et al., 2012; Irfan et al., 2014; Saidi et al., 2014; Qing et al., 2015). In addition, GPx is a powerful scavenger of H_2_O_2_ and lipid hydroperoxides with the help of GSH in plants (Lin et al., 2012; Ali et al., 2015; Qing et al., 2015). GPx is believed to be a key enzyme that can be activated by Se in various plants exposed to diverse environmental stresses (Lin et al., 2012; Ali et al., 2015; Qing et al., 2015). In the presence of Se, GPx activity was enhanced compared to the group with heavy metals only. The increase in the activity of the GPx enzyme in response to the presence of Se suggests a unique role for this enzyme in counteracting oxidative stress in plants. Selenium-induced changes in the activity of oxidoreductase enzymes have been identified in some crops (Shekhawat et al., 2010; Lin et al., 2012; Irfan et al., 2014; Saidi et al., 2014; Qing et al., 2015). Selenium has synergistic effects on the transcription of antioxidative enzymes such as copper-zinc SOD (CuZnSOD) and GPx in plants, and promotes H_2_O_2_ scavenging by increasing GPx activity, which has been initially identified as an abiotic stress responsive enzyme (Shekhawat et al., 2010; Lin et al., 2012; Irfan et al., 2014; Saidi et al., 2014; Qing et al., 2015). From this study, we found that SOD activity was lower as a whole without heavy metals. Furthermore, the effects of Se accumulation on GPx and SOD activities are positive, while the effects of Cd and Pb on GPx and SOD activities are negative. SOD and GPx activities were depressed by both Cd and Pb exposure, while in other studies on *Brassicaceae* which enhanced by the same treatments (Lin et al., 2012; Qing et al., 2015). However, it needs more data to conclude the contribution of Se to the antioxidative system of Cd and Pb toxicity in oilseed rape plant. The use of scanning electron microscopy (SEM) and transmission electron microscopy (TEM) would be helpful for determining the effect of Se on inducing ultrastructural changes of oilseed rape plant tissues to heavy metal toxicity in oilseed rape plant.

In regards to Se speciation in plants, we can provide some possible mechanisms for the interactions of Se with heavy metals. Some crops convert Se mainly into SeMet and incorporate it into protein in place of methionine (Met) (Bañuelos et al., 2011; Persson et al., 2012, 2016a,b; Winkel et al., 2012; Yuan et al., 2013). In this study, the level of Se-Met was higher in the Cd and Pb treatments than in control, which suggests the protective role of Se against Cd and Pb toxicity might depend on the competition between Se and heavy metals for binding with the functional bioligands. Furthermore, the presence of Cd and Pb in the growth medium has a great effect on Se speciation transformed by plants, which might be also considered as one of the important conditions of Se-induced Cd and Pb tolerance in oilseed rape plants. Further studies are necessary to determine that distribution and speciation of Se in oilseed rape plant cells using synchrotron-based techniques, such as X-ray fluorescence and X-ray absorption spectrometry, and mass spectrometry-based techniques, such as secondary ion mass spectrometry and laser-ablation inductively coupled plasma mass spectrometry (Zhao et al., 2014; Persson et al., 2016a,b).

In addition, crop production is greatly influenced by abiotic stresses imposed by environmental factors such as heavy metals. A thorough understanding of plant response to heavy metals stress at the molecular level is very important for its effective management (Schützendübel and Polle, 2002). Therefore, further studies are required to elucidate the molecular mechanism for Se alleviating Cd and Pb toxicity in oilseed rape plant by omics approaches such as proteomics and metabolomics (Hesketh, 2008; Debnath et al., 2011; Hesketh and Méplan, 2011; Wang et al., 2013; Sinha et al., 2016). Specifically, Se is one of essential micronutrients for animals and human health but at high concentrations, widespread use of Se fertilization needs to be carefully evaluated in the context of potential ecological impacts because Se can become toxic to vertebrates.

## Conclusion

In conclusion, the present results demonstrated that Se can reduce uptake of Cd and Pb. This effect was especially observed when the growing medium was supplemented with Se at 15 mg kg^-1^, which alleviated the negative effect of Cd and Pb on growth and led to a decrease in oxidative injuries caused by Cd and Pb. The protective effect of Se in oilseed rape (*B. napus* L.) plants subjected to Pb and Cd exposure is complex. The mechanism involved in the prevention of Cd and Pb stress is mainly linked to both the inhibition of uptake and translocation of heavy metals from the roots to shoot and the reduction of oxygen radicials. Consequently, these results suggest that Se has antioxidant properties or that Se activates protective mechanisms that can alleviate oxidative stress in both enzymatic and non-enzymatic ways. Therefore, based on our results, we propose that the protective role of Se may be related to the decrease in lipid peroxidation, the improvement of scavenging capability of ROS, and the decrease in Cd and Pb uptake, transport and accumulation in plant tissues. Furthermore, the relevant Cd and Pb detoxification mechanisms by Se might be connected to the speciation transformation to non-toxic species.

## Author Contributions

ZW and ML wrote the main manuscript text, and ZW and ML prepared all figures and tables. GB and Z-QL revised the manuscript. XY and YL have provided input and assistance to the submission of the final manuscript. All authors reviewed the manuscript.

## Conflict of Interest Statement

The authors declare that the research was conducted in the absence of any commercial or financial relationships that could be construed as a potential conflict of interest.
